# Oxidative stability and affective/descriptive sensory properties of cashew nut (*Anacardium occidentale* L.) oil during accelerated storage conditions

**DOI:** 10.1111/1750-3841.70176

**Published:** 2025-04-09

**Authors:** Amanda Rodrigues Leal, Gilleno Ferreira de Oliveira, Emilly Kaiane Maia da Silva, Ana Jady Cavalcanti Araújo, Idila Maria da Silva Araújo, Hilton César Rodrigues Magalhães, Paulo Riceli Vasconcelos Ribeiro, Arthur Claudio Rodrigues de Souza, Ana Paula Dionísio, Paulo Henrique Machado de Sousa

**Affiliations:** ^1^ Department of Food Engineering Federal University of Ceara Fortaleza Ceará Brazil; ^2^ Embrapa Agroindústria Tropical Fortaleza Ceará Brazil; ^3^ Post‐Graduate Program in Gastronomy, Culture and Art Institute Federal University of Ceará Fortaleza Ceará Brazil

**Keywords:** *Anacardium occidentale* L., Check‐All‐That‐Apply (CATA) test, cold‐pressed oil, fatty acid profile, focus group

## Abstract

**Abstract:**

Cashew nut (*Anacardium occidentale* L.) oil is not commonly consumed, but it has great potential to add value to broken nuts. Therefore, studies on its characteristics are important to provide a basis for encouraging consumption. This study aimed to characterize cashew nut oil's physical, chemical, and sensory composition. It also evaluated changes in the oil over 60 days of storage at 30°C, 40°C, and 50°C (accelerated storage). The results showed that cashew nut oil contains anacardic acids and phytosterols and is primarily composed of oleic acid (65.24%–66.49%). Throughout storage, subtle changes in the oxidative quality of the oil were observed, particularly at 50°C, with increases in acid value (0.74–0.96 mg KOH/g) and peroxide value (1.43–4.60 meq/kg), color differences (Δ*E*, 0.37–8.83), and a reduction in polyunsaturated fatty acids (16.98%–16.63%). However, the acidity and peroxide values did not exceed the limits established by the Codex Alimentarius (4.0 mg KOH/g and 15 meq/kg, respectively). Sensory acceptance decreased over time, but at the end of storage, the oil still received scores above 6 (*liked slightly*). Furthermore, the Check‐All‐That‐Apply test revealed that the oil exhibited positive sensory attributes, such as yellow color, shiny, light appearance, sweet taste, neutral flavor, and cashew nut aroma. It was concluded that the oil has great potential for commercialization and consumption, both for direct use and in culinary preparations.

**Practical Application:**

Cashew nuts are widely consumed in Brazil, but their oil remains unknown to consumers. However, this product has great potential for commercialization, as it is a food with adequate nutritional, sensory, and oxidative qualities to be included in a balanced and healthy diet, with potential applications for direct consumption and in culinary preparations. It could also help strengthen the production chain for cashew nuts with lower commercial value (broken cashew nuts). The current study aims to expand knowledge about the nutritional and sensory characteristics, as well as the oxidative stability, of cashew nut oil.

## 1 INTRODUCTION

Cashew nuts are typically consumed as snacks, either salted or unsalted, dry roasted, and in various food formulations, such as cereal bars, pastes, drinks, and cookies (Amevor et al., 2018; Carvalho et al., 2022; Lima et al., 2021; Moreira‐Araújo et al., 2021; Oladebeye & Oladebeye, 2023). Although this nut is rich in lipids, its oil is not a product commonly sold or consumed (Carvalho et al., 2022). Still, it holds great potential for adding value to nuts of lower commercial value. However, studies on its characteristics, sensory and nutritional properties, and stability are essential to provide a basis for encouraging the consumption of this product and expanding the uses of cashew nuts.

Edible oils provide essential nutrients for the human body and play a significant role in health (Deng et al., 2022). Cashew nut oil is a source of monounsaturated (MUFA) and polyunsaturated (PUFA) fatty acids, such as oleic acid (MUFA) and linoleic acid (PUFA), which are associated with cardiovascular health (Leal et al., 2023; Zanqui, Silva, Ressutte, Morais, et al., 2020). This oil also contains other health‐beneficial substances, such as polyphenols (anacardic acids), tocopherols (α, β, and γ‐tocopherol), and phytosterols (β‐sitosterol, δ‐5‐avenasterol, and campesterol) (Mattison et al., 2018; Toschi et al., 1993; Zanqui, Silva, Ressutte, Morais, et al., 2020). Furthermore, the oil exhibits sensory characteristics that are reminiscent of cashew nuts, such as yellow color, aroma, and flavor, which arouse the interest of potential consumers regarding its use in culinary preparations (Carvalho et al., 2022; Carvalho et al., 2018). Cashew nut oil also contains volatile compounds that are important in nut oils, such as pyrazines, furans, and aldehydes, which are key compounds contributing to the nutty, roasted, and sweet aromas (Hu et al., 2021; Leal et al., 2023). Additionally, it is oxidatively stable when subjected to high temperatures (Liu et al., 2023).

However, studying the shelf life of foods is essential to ensure they remain safe for consumption while retaining their suitable characteristics. Lipid oxidation is the primary factor that triggers the deterioration of oils (El‐Hadary & Taha, 2020). Monitoring oxidation reactions under normal shelf like conditions can be time‐consuming and take months. Therefore, methodologies for accelerated shelf like studies have been developed by altering the storage environment, such as temperature, light, and the concentration of pro‐oxidant compounds. Temperature is one of the most critical factors affecting food reaction kinetics, which is why elevated temperatures are commonly used in accelerated storage studies (Conte et al., 2020; Hoppenreijs et al., 2021). During the storage period of edible oils, lipid oxidation can be monitored through acidity and peroxide indices, with limits established by the Codex Alimentarius (2021), as well as more advanced methodologies, such as sensory and fatty acid analyses (Martín‐Torres et al., 2023).

Based on the above, the objective of the present study was to characterize cashew nut oil regarding its physical, chemical, and sensory composition and to evaluate the changes that occurred over 60 days of storage at 30°C (normal storage temperature), as well as at 40°C and 50°C (accelerated conditions).

## 2 MATERIALS AND METHODS

### 2.1 Raw material

Cashew nuts (P1—large pieces, first quality) (Association of Food Industries—AFI, 2016) were used, as per the study results by Leal et al. (2023). All samples were vacuum‐packed in bags that protected them from direct light and stored at −18°C until processing to obtain cashew nut oil.

### 2.2 Reagents

All chemicals were of analytical grade or higher and were purchased from Sigma‐Aldrich. Aqueous solutions were prepared with water purified by a Milli‐Q System (Millipore Lab.).

### 2.3 Cashew nut oil processing

Cashew nuts were roasted at 110°C for 15 min in a forced convection air oven (Marconi MA035). Subsequently, the samples were pressed in a hydraulic press (Hidraumon PH30) under the following conditions: an area of 177 cm^2^, a pressure of 100 kgf/cm^2^, and a temperature of 30°C. The oil obtained was centrifuged at room temperature for 30 min at 4500 rpm in a Thermo Scientific Heraeus Megafuge 40 centrifuge.

After processing, the oil samples were placed in 50‐mL transparent glass bottles with metal lids. Glass containers were used to store the samples because they were impermeable and inert, providing better oil preservation. In contrast, plastic is more permeable to oxygen and light, intensifying hydrolytic reactions (Méndez & Falqué, 2007).

The samples were separated and stored at 30°C, 40°C, and 50°C for 60 days without light in Biochemical Oxygen Demand (BOD) incubators (model TE‐371, Tecnal). Every 15 days, flasks stored at each temperature were removed and stored at −80°C until analysis, resulting in intervals of 0, 15, 30, 45, and 60 days.

Samples were processed in triplicate.

### 2.4 Cashew nut oil characterization

#### 2.4.1 Anacardic acids

The analysis and identification of compounds were performed according to Alves et al. (2023) and Cunha et al. (2017), with minor modifications. An Acquity UPLC system (Waters) was coupled to a mass spectrometer (Q‐TOF, Waters). A Waters Acquity BEH C18 column was used for separation (150 mm × 2.1 mm × 1.7 µm) and set at 40°C. An injection volume of a 5‐µL aliquot of each extract was subjected to an exploratory gradient with the mobile phase composed of deionized water (A) and acetonitrile (B), both containing formic acid (0.1% v/v). The extracts were subjected to the following exploratory gradients: 2%–100% B (22.0 min), 100% B (22.1–25.0 min), and 2% B (26.0–30 min), with a flow rate of 0.3 mL/min. Ionization was performed using an electrospray ionization source in positive mode, acquired in the 110–1180 Da range. The optimized instrumental parameters were as follows: capillary voltage at 3.2 kV, cone voltage at 15 V, source temperature at 120°C, desolvation temperature at 350°C, and desolvation gas flow at 500 L/h. The system was controlled using MassLynx software (Waters Corporation).

#### 2.4.2 Phytosterols

The phytosterols were determined according to the methodology of Beveridge et al. (2002). The compounds were derivatized using N‐O‐bis(trimethylsilyl) trifluoroacetamide (BSTFA; Sigma‐Aldrich Chemical Co.). Analyses were performed in triplicate, using 2 µL for each injection, with a split ratio of 1:10. The quantification of phytosterols was conducted using the 5α‐cholestane standard (Sigma‐Aldrich Chemical Co.) (Beveridge et al., 2002). The gas chromatography‐mass spectrometry analysis was performed using an Agilent model 7890B, equipped with a mass spectrometer detector, model 5977A. The chromatographic column used was RTX 5 (30 m length, 0.25 mm internal diameter, and 0.25 µm film thickness). The temperature program was as follows: a 10°C/min ramp from 120°C to 275°C, remaining at 275°C for 50 min. The interface temperature between the chromatograph and the selective mass detector was 280°C, and ionization was performed by electron impact (70 eV) with the ion source maintained at 150°C.

### 2.5 Oxidative stability of cashew nut oil during accelerated storage

Analyses of acid value, peroxide value, color, and fatty acid profile were carried out on all oil samples obtained, stored at temperatures of 30°C, 40°C, and 50°C, and for 0, 15, 30, 45, and 60 days.

#### 2.5.1 Acid and peroxide values

The acid and peroxide values were measured using the official methods Ca 5a‐40 and Cd 8–53 (AOCS, 2003). Approximately 5 g of the sample was weighed in triplicate.

#### 2.5.2 Color analysis

Color analyses were performed by measuring the direct reflectance on the rectangular coordinate system (L*, brightness; a*, intensity of red and green; b*, intensity of yellow and blue) using the CIELAB color scale with a colorimeter (CM‐5, Konica Minolta). The results were also expressed as the color differential (Δ*E*), calculated according to Equation (1) (Mokrzycki & Tatol, 2011):

(1) $$\Delta E={\left({\left(\Delta L^{*}\right)}^{2}+{\left(\Delta a^{*}\right)}^{2}+{\left(\Delta b^{*}\right)}^{2}\right)}^{1/2}$$

Analyses were performed in triplicate.

#### 2.5.3 Fatty acid profiling of cashew nut oil by gas chromatography‐flame ionization detector

The fat samples previously extracted were converted to fatty acid methyl esters (FAMEs) following the procedure described by Hartman and Lago (1973) and Milinsk et al. (2011) to determine the fatty acid profile of cashew nut oil. An amount of 100 mg of oil was mixed with 3.0 mL of hexane and 4.0 mL of 0.50 mol L^−^¹ sodium hydroxide in methanol, and the mixture was heated under reflux for 5 min at 70°C. After adding 5.0 mL of the esterification reagent (prepared from a mixture of 10.0 g of ammonium chloride, 300.0 mL of methanol, and 15.0 mL of sulfuric acid), 4.0 mL of sodium chloride and 3.0 mL of hexane, the mixture was shaken for 30 s. The organic phase was collected, and the solvent was evaporated. The methyl esters were solubilized in hexane before injection into the gas chromatograph.

After extraction, the materials were analyzed using gas chromatography (GC), equipped with an FID (GC2010 Plus, Shimadzu) and a stationary phase bis‐cyanopropyl‐polydimethylsiloxane capillary column (SP2560, 100 m × 0.25 mm, *df* 0.20 µm; Supelco®). The column temperature was programmed as follows: it was initially maintained at 80°C, then increased by 11°C min^−^¹ until 180°C, then by 5°C min^−^¹ until 220°C, and held for 23 min. Hydrogen was the carrier gas at a 1.5 mL min^−^¹ flow rate. The separation ratio was 1:30, with the injector and detector temperatures set at 220°C. Assays were performed in triplicate. The FAMEs were identified by comparing the retention times with those of a previously injected fatty acid standard mix (code CRM47885, Supelco). The contribution of each compound to the mixture was calculated by the relative area (%) of its respective peak in the chromatogram. Fatty acids were reported by their common names.

#### 2.5.4 Affective and descriptive sensory properties of cashew nut oil during accelerated storage

Sensory tests were conducted on samples at three temperatures (30°C, 40°C, and 50°C) and intervals of 0, 30, and 60 days. The trials were carried out through a focus group and the application of a questionnaire containing a hedonic scale and the Check‐All‐That‐Apply (CATA) test.

The panel consisted of untrained volunteers aged between 18 and 40 years. All the participants were academics or had academic training in food, including students and professionals in nutrition, gastronomy, and food engineering: the group comprised six men and seven women, all frequent consumers of vegetable oils.

Thirteen participants participated in the tests, divided into two sessions: seven volunteers participated in the first session, and the remaining six participated in the second. Dias (2000) states that focus groups with six to ten people are recommended to promote a discussion that encourages everyone's participation and interaction in a relatively orderly way. Each session lasted an average of 90 min and was led by a moderator with two assistants. First, 10 mL of each sample was presented monadically to each panelist in a white porcelain cup with a 50 mL capacity. Subsequently, sheets containing the hedonic scale and CATA questions were distributed. Finally, a focus group discussion was held so that tasters could share their impressions of the samples evaluated.

The study was reviewed and approved by the Research Ethics Committee of the Federal University of Ceará (Reference number 4.729.905), and informed consent was obtained from each participant before their involvement in the study.

#### 2.5.5 Acceptance testing

Sensory acceptance was evaluated using a form with a structured hedonic scale of nine categories, where 1 represents the lowest score (*disliked extremely*) and 9 represents the highest (*liked extremely*) (Stone & Sidel, 2004). This scale indicated the degree to which the panelists liked or disliked the samples concerning color, appearance, aroma, flavor, and overall impression. These attributes were selected to provide an overview of the consumers’ responses to the key attributes considered important for assessing the quality of cashew nut oil.

##### CATA test

In the same session, the CATA test was conducted to characterize the product using a list of sensory descriptors, where panelists were asked to select all the terms that applied to the sample (Ares et al., 2014). A form containing sensory descriptors, selected from a study by Carvalho et al. (2022) with modifications based on preliminary tests (Ares et al., 2014), was provided, where panelists were required to indicate which terms applied to the oil samples.

Piochi et al. (2021) reported that the CATA test was effective in detecting sensory changes in olive oil during storage at room temperature, making it a promising technique for shelf life studies, as it is a quick test that generates important descriptive information about the products.

##### Focus group

The focus group was based on the methodology described by Lawless and Heymann (2010) and aimed to evaluate consumer opinions regarding cashew nut oil samples. Volunteers were invited to participate in two sessions, each lasting an average of 60 min. In each session, panelists received 10 mL of the samples in a white porcelain cup with a 50 mL capacity. They were instructed to assess the sample's color, appearance, aroma, and flavor using a piece of white bread. Additionally, questions were asked about the oil's market availability, healthiness, consumption, use in culinary preparations and food products, packaging, and market value.

An assistant took notes and recorded the focus group sessions. The data collected were evaluated to assist in the sensory characterization of the samples.

### 2.6 Statistical analysis

The data obtained from chemical, physicochemical, and acceptance analyses were submitted to analysis of variance with a significance level of 5% to identify significant differences between the means.

Cochran's *Q* test was performed on the means of the CATA results to identify significant differences between the samples for each term of the CATA questionnaire (*p* ≤ 0.05). The McNemar test was also applied to determine which samples differed significantly at the 5% significance level. Correspondence analysis (CA) was performed to observe the proximity between samples and attributes, where samples that were spatially close to each other were considered similar, and samples close to an attribute indicated high percentages of that attribute being selected. A penalty analysis (PA) was applied between the attributes and global acceptance to identify the positive and negative factors influencing the acceptance of the samples. PA provided a graph representing the average impact of attributes on taste (positive or negative) on the *Y*‐axis and the frequency of attribute selection in the samples on the *X*‐axis. A vertical dotted line on the *X*‐axis represented the threshold above which results were considered significant (20%). All analyses were performed using XLSTAT 2023 (Lumivero).

## 3 RESULTS AND DISCUSSION

### 3.1 Cashew nut oil characterization: Anacardic acids

**FIGURE 1:**
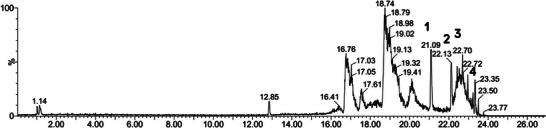
Chromatogram and identification of anacardic acids in cashew nut oil.

The anacardic acids identified in cashew nut oil were 15:3, 15:2, 17:3, and 15:1 (Figure 1; Table 1). Similar results were reported in studies conducted with cashew nut oils. Mattison et al. (2018) identified anacardic acids C15:3, C15:2, and C15:1, whereas Gómez‐Caravaca et al. (2010) reported the presence of anacardic acids 15:0, 15:1, 15:2, and 15:3 in cashew nut oil.

**TABLE 1:** Identification of anacardic acids in cashew nut oil.

Peak	Rt (min)	[M‐H]^−^ observed	[M‐H]^−^ calculated	Anacardic acid
1	21.08	341.2119	341.2117	15:3
2	22.12	343.2272	343.2273	15:2
3	22.57	369.2426	369.2430	17:3
4	23.33	345.2426	345.2430	15:1

Abbreviation: Rt, retention time.

Studies suggest that anacardic acids possess several biological properties, such as anti‐inflammatory (Önal et al., 2020), antimicrobial (Lima et al., 2020), antidepressant (Gomes Júnior et al., 2019), and antitumor (Galot‐Linaldi et al., 2021) effects, as well as improved bone density (Zhao et al., 2019). Furthermore, anacardic acids can reduce lipid oxidation in foods by inhibiting the action of lipoxygenase (LOX) enzymes. LOX enzymes are a family of enzymes that catalyze the oxidation of polyunsaturated fatty acids, such as linoleic acid. These enzymes are present in a wide variety of plant‐ and animal‐based foods, including vegetable oils, and can lead to the development of rancid or unpleasant flavors (Aprea et al., 2018; Ha & Kubo, 2005; Wisastra et al., 2012).

### 3.2 Cashew nut oil characterization: Phytosterols

The phytosterols identified in cashew nut oil were β‐sitosterol, cycloartenol, lanosterol, and campesterol (Table 2). The literature on phytosterols in cashew nut oil is scarce. However, Zanqui, Silva, Ressutte, Morais, et al. (2020) reported sitosterol levels ranging from 373.2 to 719.2 mg/kg. In turn, Ryan et al. (2006) quantified 1768.0 mg/kg of β‐sitosterol, 105.3 mg/kg of campesterol, and 116.7 mg/kg of stigmasterol. Griffin and Dean (2017) found β‐sitosterol (1521 mg/kg), campesterol (142 mg/kg), stigmasterol (10 mg/kg), and brassicasterol (7.5 mg/kg) in cashew nut oil.

**TABLE 2:** Phytosterol content in cashew nut oil.

Phytosterol	mg/kg
Campesterol	27.82 ± 5.2
β‐Sitosterol	497.65 ± 111.6
Lanosterol	53.88 ± 9.6
Cycloartenol	90.84 ± 22.7

*Note*: Results are expressed in mean and standard deviation.

Concerning other seeds, a study by Giuffrè and Capocasale (2016) found that tomato seed oil had β‐sitosterol levels ranging from 938 to 1088 mg/kg and campesterol levels between 5.01 and 5.36 mg/kg. On the other hand, Grajzer et al. (2020) reported a β‐sitosterol content of 172.3 mg/kg in pumpkin seed oil, and no campesterol was detected in the sample analyzed. This indicates that cashew nut oil has intermediate levels of β‐sitosterol and higher levels of campesterol than those mentioned above.

Phytosterols significantly reduce serum levels of total cholesterol and low‐density lipoprotein, as their structure is similar to cholesterol, thus competing for intestinal absorption (Feng et al., 2020; Moumen et al., 2015).

### 3.3 Oxidative stability of cashew nut oil during accelerated storage: Acid, peroxides, and color

The acid and peroxide values of cashew nut oil ranged from 0.74 (T0) to 0.96 (T60/50) and from 1.43 (T0) to 4.60 (T60/50), respectively (Table 3). There was a gradual increase in storage time, particularly at 50°C (Table 3; Table S1). However, the maximum allowable values of 4.0 mg KOH/g for acidity and 15 meq/kg for peroxide were not reached (Codex Alimentarius, 2021). This result corroborates several studies that analyzed the stability of vegetable oils under accelerated conditions and at room temperature (Elouafy et al., 2022; Rabiej‐Kozioł et al., 2021; Shen et al., 2020).

**TABLE 3:** Acid, peroxide, and color values of cashew nut oil stored at 30°C, 40°C, and 50°C for 60 days.

Temperature (°C)	Time (days)	Sample	Acid value (mg KOH/g)	Peroxide value (meq/kg)	L*	a*	b*	∆*E*
30	0	T0	0.74 ± 0.09	1.43 ± 0.11	94.91 ± 0.94	−3.50 ± 0.25	43.85 ± 0.35	
	15	T15/30	0.78 ± 0.12	1.58 ± 0.02	94.83 ± 0.60	−3.32 ± 0.18	43.53 ± 0.64	0.37
	30	T30/30	0.85 ± 0.08	1.57 ± 0.15	94.38 ± 2.26	−3.51 ± 0.32	42.63 ± 0.52	1.33
	45	T45/30	0.74 ± 0.09	1.47 ± 0.09	95.84 ± 1.54	−3.46 ± 0.38	42.91 ± 0.27	1.33
	60	T60/30	0.80 ± 0.07	1.75 ± 0.21	89.00 ± 6.98	−2.70 ± 0.55	43.91 ± 0.98	5.96
40	0	T0	0.74 ± 0.09	1.43 ± 0.11	94.91 ± 0.94	−3.50 ± 0.25	43.85 ± 0.35	
	15	T15/40	0.86 ± 0.13	1.74 ± 0.11	93.97 ± 1.20	−3.38 ± 0.34	42.74 ± 0.53	1.46
	30	T30/40	0.93 ± 0.09	1.86 ± 0.07	93.65 ± 1.38	−3.35 ± 0.14	42.23 ± 0.56	2.06
	45	T45/40	0.76 ± 0.11	2.03 ± 0.24	92.20 ± 4.79	−3.25 ± 0.57	41.25 ± 0.68	3.76
	60	T60/40	0.88 ± 0.08	2.44 ± 0.24	83.98 ± 1.35	−0.83 ± 0.85	43.81 ± 1.56	11.25
50	0	T0	0.74 ± 0.09	1.43 ± 0.11	94.91 ± 0.94	−3.50 ± 0.25	43.85 ± 0.35	
	15	T15/50	0.85 ± 0.09	1.87 ± 0.17	94.33 ± 2.51	−3.37 ± 0.56	42.48 ± 0.08	1.49
	30	T30/50	0.96 ± 0.12	2.60 ± 0.12	91.78 ± 2.74	−2.90 ± 0.62	41.90 ± 0.32	3.74
	45	T45/50	0.88 ± 0.11	3.56 ± 0.36	91.75 ± 3.05	−2.47 ± 0.83	41.64 ± 0.59	3.99
	60	T60/50	0.96 ± 0.10	4.60 ± 0.24	86.55 ± 3.17	−1.47 ± 0.78	41.87 ± 0.74	8.83

*Note*: Results are expressed in mean and standard deviation.

Lima et al. (2014) evaluated the shelf life of cashew nut oil extracted by cold pressing and stored at 28°C for 230 days. The authors observed acidity values around 0.75–1.40 mg KOH/g and peroxide values ranging from 0.00 to 10.00 meq/kg at the beginning and end of the storage period. This shows that the oil remained within the limits established by Codex Alimentarius (2021) throughout the analyzed period.

The acidity and peroxide values are important indicators for quantifying free fatty acids and primary oxidation products of oils, respectively. In the presence of light, oxygen, and metal ions, hydrolysis of triacylglycerols occurs, leading to the release of free fatty acids, which favors oil oxidation (An et al., 2021; Zhang et al., 2020).

L*, a*, and b* values decreased as storage time progressed, especially at 50°C (Table 3; Table S1). There was also a gradual increase in the color difference (Δ*E*) compared to the control sample (T0) over time. In research on extra virgin olive oil by Iqdiam et al. (2020), the L* and b* parameters decreased over 12 months of storage at room temperature (25°C–28°C), whereas the a* parameter increased. Özcan et al. (2022) observed a reduction in extra virgin olive oil's L*, a*, and b* values during 6 months of storage at room temperature. The authors suggest that oxidation and light exposure may have caused these changes. Among the effects of lipid oxidation are the degradation and formation of colored compounds (Bayram & Decker, 2023; Chen & Sun, 2023).

### 3.4 Oxidative stability of cashew nut oil during accelerated storage: Fatty acids

The predominant fatty acids in cashew nut oils were oleic (65.24%–66.49%), linoleic (15.85%–16.90%), palmitic (8.21%–8.75%), and stearic (7.53%–8.74%) (Table 4). Similar results were obtained in a previous study on cashew nut oil obtained from nuts of different classifications, where the prevalent fatty acids were oleic (63.33%–65.28%), linoleic (16.60%–18.23%), palmitic (8.89%–9.24%), and stearic (7.08%–8.25%) (Leal et al., 2023). Liu et al. (2023) reported approximate values for oleic (60.87%), linoleic (17.33%), stearic (10.93%), palmitic (9.85%), arachidonic (0.76%), and palmitoleic (0.26%) fatty acids in cashew nut oil.

**TABLE 4:** Fatty acid composition of cashew nut oil stored at 30°C, 40°C, and 50°C for 60 days.

Temperature (°C)	Time (days)	Sample	C16:0	C16:1	C17:1	C18:0	C18:1 w9	C18:2 w6	C20:4 w6	Unidentified	SFA	MUFA	PUFA
30	0	T0	8.68 ± 0.03	0.20 ± 0.03	0.05 ± 0.04	8.60 ± 0.21	65.31 ± 0.37	16.88 ± 0.61	0.11 ± 0.04	0.16 ± 0.01	17.29 ± 0.18	65.57 ± 0.38	16.98 ± 0.57
	15	T15/30	8.58 ± 0.04	0.25 ± 0.04	0.08 ± 0.00	8.65 ± 0.28	65.31 ± 0.07	16.90 ± 0.32	0.06 ± 0.02	0.20 ± 0.03	17.23 ± 0.23	65.64 ± 0.11	16.96 ± 0.29
	30	T30/30	8.75 ± 0.09	0.30 ± 0.00	0.15 ± 0.07	8.62 ± 0.19	65.24 ± 0.19	15.99 ± 0.16	0.56 ± 0.16	0.39 ± 0.22	17.37 ± 0.10	65.69 ± 0.12	16.55 ± 0.00
	45	T45/30	8.21 ± 0.10	0.33 ± 0.06	0.27 ± 0.05	7.53 ± 0.02	66.41 ± 1.13	15.98 ± 0.92	0.80 ± 0.10	0.45 ± 0.12	15.74 ± 0.08	67.02 ± 1.02	16.79 ± 0.82
	60	T60/30	8.51 ± 0.02	0.25 ± 0.02	0.11 ± 0.07	8.47 ± 0.20	65.89 ± 0.17	16.04 ± 0.46	0.62 ± 0.37	0.12 ± 0.00	16.98 ± 0.18	66.25 ± 0.27	16.65 ± 0.09
40	0	T0	8.68 ± 0.03	0.20 ± 0.03	0.05 ± 0.04	8.60 ± 0.21	65.31 ± 0.37	16.88 ± 0.61	0.11 ± 0.04	0.16 ± 0.01	17.29 ± 0.18	65.57 ± 0.38	16.98 ± 0.57
	15	T15/40	8.57 ± 0.14	0.24 ± 0.03	0.07 ± 0.04	8.43 ± 0.02	65.65 ± 0.46	16.59 ± 0.57	0.26 ± 0.17	0.19 ± 0.11	17.00 ± 0.16	65.96 ± 0.45	16.85 ± 0.40
	30	T30/40	8.33 ± 0.11	0.37 ± 0.26	0.24 ± 0.25	8.25 ± 0.02	65.81 ± 0.09	15.85 ± 1.54	0.66 ± 0.78	0.50 ± 0.48	16.58 ± 0.14	66.42 ± 0.42	16.51 ± 0.76
	45	T45/40	8.52 ± 0.15	0.28 ± 0.02	0.05 ± 0.01	8.50 ± 0.61	65.91 ± 0.87	16.08 ± 0.56	0.50 ± 0.41	0.16 ± 0.01	17.02 ± 0.76	66.24 ± 0.91	16.58 ± 0.14
	60	T60/40	8.40 ± 0.00	0.27 ± 0.03	0.17 ± 0.12	7.88 ± 0.80	65.68 ± 0.43	16.80 ± 0.54	0.58 ± 0.33	0.23 ± 0.20	16.28 ± 0.80	66.12 ± 0.28	17.38 ± 0.88
50	0	T0	8.68 ± 0.03	0.20 ± 0.03	0.05 ± 0.04	8.60 ± 0.21	65.31 ± 0.37	16.88 ± 0.61	0.11 ± 0.04	0.16 ± 0.01	17.29 ± 0.18	65.57 ± 0.38	16.98 ± 0.57
	15	T15/50	8.58 ± 0.13	0.21 ± 0.05	0.11 ± 0.04	8.74 ± 0.36	65.39 ± 0.08	16.12 ± 0.39	0.69 ± 0.12	0.17 ± 0.09	17.32 ± 0.49	65.71 ± 0.10	16.80 ± 0.51
	30	T30/50	8.41 ± 0.07	0.24 ± 0.01	0.12 ± 0.09	8.20 ± 0.39	66.33 ± 0.16	15.93 ± 0.51	0.60 ± 0.05	0.16 ± 0.05	16.62 ± 0.32	66.70 ± 0.08	16.52 ± 0.46
	45	T45/50	8.27 ± 0.45	0.25 ± 0.10	0.14 ± 0.09	7.77 ± 0.18	66.49 ± 0.07	15.94 ± 0.20	0.84 ± 0.37	0.30 ± 0.18	16.04 ± 0.62	66.88 ± 0.27	16.78 ± 0.17
	60	T60/50	8.59 ± 0.10	0.24 ± 0.01	0.10 ± 0.01	8.33 ± 0.44	65.92 ± 0.26	16.23 ± 0.27	0.40 ± 0.33	0.20 ± 0.02	16.91 ± 0.33	66.26 ± 0.25	16.63 ± 0.06

*Note*: Results are expressed in mean and standard deviation.

Abbreviations: MUFA, monounsaturated fatty acids; PUFA, polyunsaturated fatty acids; SFA, saturated fatty acids.

When comparing the fatty acid profile of cashew nut oil with other edible oils, similarities were observed with peanut oil, which is mainly composed of oleic acid (44.61%–50.95%), followed by linoleic acid (29.92%–35.17%), palmitic acid (8.42%–10.90%), and stearic acid (2.11%–3.95%), with saturated fatty acid (SFA) contents ranging from 16.56% to 20.84%, MUFA from 45.53% to 51.87%, and PUFA from 30.01% to 35.35% (Giuffrè et al., 2016). Sesame oil, unlike cashew nut oil, has linoleic acid (42.1%) as its main fatty acid, followed by oleic acid (41.9%), palmitic acid (11.3%), and stearic acid (4.9%), with SFA contents of 16.3% and unsaturated fatty acids (UFA) making up 84.3% (Gharby et al., 2017). Sunflower oil also differs in its fatty acid profile, with higher proportions of linoleic acid (64.64%) followed by oleic acid (23.30%) and a PUFA content of 74.73% (Nakonechna et al., 2024).

Storage time and temperature influenced the fatty acid composition of the oil samples (Table 4; Table S2). Over time, the proportions of saturated fatty acids (palmitic acid, stearic acid, and SFA) decreased, while monounsaturated fatty acids (palmitoleic acid, heptadecenoic acid, oleic acid, and MUFA) increased, and polyunsaturated fatty acids (linoleic acid and PUFA) decreased, except for arachidonic acid, which increased. The reduction in polyunsaturated fatty acids indicates a certain degree of lipid oxidation during storage (Mansour et al., 2022). These results corroborate the observations made for acid and peroxide values, which increased throughout storage. In parallel, there was a reduction in the color parameters L*, a*, and b* over time. Thus, both results indicate that the oil has oxidized. Shen et al. (2020) observed that accelerated storage at 60°C significantly increased the percentages of SFA and MUFA but decreased that of PUFA in algae oil. This occurs because PUFA is more susceptible to oxidation due to more oxidizable allylic hydrogens adjacent to the double bond (Hoppenreijs et al., 2021).

Despite this change in the fatty acid profile, studies show that cashew nut oil is stable. Oliveira (2022) evaluated the oxidative stability of cashew nut oil using the Rancimat equipment, resulting in an induction period of 8.51 ± 1.01 h. Therefore, this oil is stable compared to other nut oils and extra virgin olive oil.

### 3.5 Affective and descriptive sensory properties of cashew nut oil during accelerated storage

#### 3.5.1 Focus group

In general, tasters expressed that they liked cashew nut oil and found the product interesting because it was obtained from a regional raw material (produced in northeastern Brazil). The appearance was described as “shiny,” “clear,” “light,” “fluid,” and “less viscous than extra virgin olive oil.” Its flavor, aroma, and color were highly appreciated by the panel, who stated that these characteristics caught their attention. The flavor was described as “pleasant to the palate,” “sensorially interesting,” “sweet,” “without bitterness,” “neutral,” “cashew nut‐like,” and “roasted cashew nut flavor.” Furthermore, tasters reported that the oil had aromas of both cashew nuts and roasted ones.

The oil was considered healthy because it is derived from cashew nuts, contains antioxidants, improves the health profile, benefits heart health, and has an excellent nutritional appeal. The tasters’ perspective may have been influenced by pre‐existing knowledge regarding the recognized dietary properties of cashew nuts. As highlighted in this study, cashew nut oil contains compounds such as anacardic acids (15:3, 15:2, 15:1, and 17:3), phytosterols (mainly β‐sitosterol), and mono‐ and polyunsaturated fatty acids like oleic and linoleic acid (Sections 3, 3.1, and 3.4, respectively). Research shows that monounsaturated and polyunsaturated fatty acids are anti‐inflammatory and help prevent chronic and cardiovascular diseases (Uslu & Özcan, 2019; Zanqui, Silva, Ressutte, Morais, et al., 2020). Moreover, compounds like phytosterols and anacardic acids provide additional health benefits (Carvalho et al., 2018; Zanqui, Silva, Ressutte, Rotta, et al., 2020).

The main suggestion for consuming the oil was its direct use, added to salads or even spread on bread (like butter). Another proposition was incorporating it into pesto sauce, breads, cakes, biscuits, and farofas preparation*s*. However, the panelists stated that, sensorially, the oil had the most potential for application in sweet preparations. This result was similar to findings by Carvalho et al. (2022), where the flavor of both cold‐ and hot‐pressed cashew nut oil was considered pleasant for direct consumption, such as in salads. Oliveira (2022) noted that cashew nut oil has good oxidative stability for use in preparations subjected to moderate heat, as its smoke point is higher than 180°C.

Furthermore, this study corroborated the suggestion of using the oil in sweet preparations. Good sensory acceptance was observed for a cream made with cashew nut oil, sugar, cocoa, and cashew nuts. The acceptance scores for global acceptance, appearance, aroma, flavor, and texture ranged from *liked moderately* to *liked very much*.

Finally, the panel expressed concern about the possible product price, as it is derived from a food they already consider expensive, cashew nuts. In light of this, a suggestion was made for the oil to be sold in 200‐ to 250‐mL bottles to make the price more accessible to potential consumers. They indicated they would be willing to pay amounts equal to or slightly higher than for extra virgin olive oil. The study by Carvalho et al. (2022) also highlighted the price of cashew nut oil as a significant consideration for its market introduction. The preferred packaging material was glass, but the color was a point of contention, as oxidation had to be considered. Therefore, dark glass would be a suitable choice. However, the transparent glass would be more attractive, as it would showcase the yellow color of the oil and its shiny, clear appearance. Based on this, transparent packaging is considered more appropriate, as this product is still unfamiliar to potential consumers. In this way, the color and appearance of cashew nut oil would be visible during its market display.

#### 3.5.2 Acceptance test

Cashew nut oil obtained average ratings for appearance and aroma between *liked moderately* and *liked very much* and for flavor and overall acceptance between *liked slightly* and *liked very much* (Table 5). Oliveira (2022) reported in his study that cashew nut oil received values ranging from *liked moderately* to *liked very much* for the attributes of global acceptance, appearance, and flavor and between *liked slightly* and *liked moderately* for the aroma attribute. However, when the results were evaluated in terms of acceptance ranges, it was observed that 88% of tasters accepted cashew nut oil, with scores ranging from 6 (*liked slightly*) to 9 (*liked very much*) (Oliveira, 2022).

**TABLE 5:** Average acceptance of cashew nut oil stored at 30°C, 40°C, and 50°C for 60 days.

Temperature (°C)	Time (days)	Sample	Appearance	Aroma	Flavor	Global acceptance
30	0	T0	8.00	7.85	7.85	7.69
	30	T30/30	7.50	7.50	7.36	7.36
	60	T60/30	7.08	7.23	7.33	7.31
40	0	T0	8.00	7.85	7.85	7.69
	30	T30/40	7.42	7.38	7.31	7.31
	60	T60/40	7.00	7.00	7.31	7.23
50	0	T0	8.00	7.85	7.85	7.69
	30	T30/50	7.31	7.15	7.09	7.25
	60	T60/50	7.15	7.33	6.67	6.69

The sensory acceptance parameters (color, flavor, aroma, and overall impression) underwent significant changes throughout storage (Table S3). Nonetheless, even at the end of the stability period, the samples were still well accepted, with global acceptance scores ranging from 6 to 7 (*liked slightly* to *liked moderately*), suggesting that the oil could be stored for longer than 60 days, even at a temperature of 50°C (Table 5).

Research indicates that the sensory acceptance of sunflower oil gradually decreased over time, eventually leading to its rejection after 12–30 days of storage at 65°C (Abdelli et al., 2022; Yang et al., 2023; Meng, Wang, Dong, Chen & Wang, 2021). This is likely due to the lipid composition of sunflower oil, which has a higher proportion of polyunsaturated fatty acids, particularly linoleic acid (around 69%) (Meng et al., 2021), resulting in lower oxidative stability compared to cashew nut oil, which is mainly composed of oleic acid (approximately 65%), a monounsaturated fatty acid (Leal et al., 2023). In this study, up to 66.49% oleic acid was found in the cashew nut oil samples (Section 3.4), supporting the references mentioned above, which likely contributed significantly to the oxidative stability of the oil under investigation.

Studies using the Rancimat oxidative stability measurement equipment show that cashew nut oil is relatively stable, with an induction period of 46 h at 100°C in a 10 L/h airflow (Carvalho et al., 2018), while sunflower oil exhibited an induction period of approximately 8 h at 110°C with a 15 L/h airflow (Abdelli et al., 2022).

#### 3.5.3 CATA test

Table 6 presents the results of the CATA questions for each attribute. Higher proportions (close to 1.00) indicate that the tasters selected the term more frequently (Pramudya & Seo, 2018). Thus, the attributes most commonly used to describe the cashew nut oil samples were “yellow color,” “shiny,” “translucent,” “greasy aspect,” “roasted cashew nut aroma,” “sweet taste,” “roasted cashew nut flavor,” and “cashew nut aftertaste” (Table 6). The most frequently used terms best describe the positive or negative samples and are relevant for product optimization (Leal et al., 2021). On the other hand, the least frequently attributed descriptors were “turbid,” “rancid aroma,” “acidic taste,” “bitter taste,” “fruity flavor,” and “rancid flavor.” A sensory study on cold‐ and hot‐pressed cashew nut oils reported that the most commonly used terms to describe the samples were “cashew nut flavor and aroma,” “yellow color,” and “brightness.” The least used terms were “turbid,” “rancid odor and flavor,” “roasted flavor,” and “bitter taste” (Carvalho et al., 2022).

**TABLE 6:** Check‐All‐That‐Apply (CATA) test data by frequency of selected terms referring to cashew nut oil samples stored at 30°C, 40°C, and 50°C for 60 days.

Attributes	T0	T30/30	T30/40	T30/50	T60/30	T60/40	T60/50	*p* values
Yellow color	1.00a	1.00a	1.00a	1.00a	1.00a	1.00a	1.00a	1.000
Shiny	1.00a	0.92a	0.92a	1.00a	0.92a	1.00a	1.00a	0.677
Turbid	0.08a	0.23a	0.23a	0.39a	0.15a	0.31a	0.15a	0.387
Translucent	0.85a	0.69a	0.77a	0.69a	0.77a	0.62a	0.77a	0.726
Presence of particles	0.15a	0.31a	0.23a	0.46a	0.69a	0.39a	0.23a	0.020*
Greasy aspect	0.69a	0.69a	0.85a	0.69a	0.69a	0.62a	0.62a	0.484
Aroma of CN *in natura*	0.31a	0.39a	0.23a	0.31a	0.39a	0.46a	0.46a	0.715
Roasted CN aroma	0.85a	0.85a	1.00a	0.85a	0.77a	0.77a	0.85a	0.668
Rancid aroma	0.00a	0.00a	0.08a	0.15a	0.08a	0.08a	0.23a	0.086
Sweet taste	0.62a	0.62a	0.46a	0.54a	0.62a	0.62a	0.46a	0.750
Salty taste	0.39a	0.39a	0.46a	0.39a	0.23a	0.46a	0.31a	0.477
Acidic taste	0.00a	0.08a	0.00a	0.08a	0.00a	0.31a	0.23a	0.029*
Bitter taste	0.00a	0.08a	0.00a	0.31a	0.15a	0.23a	0.23a	0.122
Flavor of CN *in natura*	0.39a	0.39a	0.23a	0.39a	0.39a	0.46a	0.46a	0.731
Roasted CN flavor	0.77a	0.85a	1.00a	0.69a	0.77a	0.62a	0.85a	0.231
Fruity flavor	0.15a	0.15a	0.08a	0.08a	0.08a	0.08a	0.23a	0.313
Rancid flavor	0.00a	0.15a	0.08a	0.15a	0.23a	0.15a	0.23a	0.238
CN aftertaste	0.69a	0.62a	0.77a	0.62a	0.62a	0.92a	0.77a	0.217

*Note*: Means with the same letters and on the same line do not show significant differences according to McNemar's procedure (*p* ≤ 0.05).

Abbreviation: CN, cashew nut.

*Significant difference in CATA terms as a function of oil storage time and temperature according to Cochran's *Q* test (*p* ≤ 0.05).

These results corroborate the data found for color, acidity, and anacardic acids. In the instrumental analysis, the color was in the yellow zone, evidenced by positive b* values (41.25–43.85), as seen in Table 3. The acidity and peroxide values observed in the samples were below the parameters required by the Codex Alimentarius (Section 3.3; Table 3), which may have contributed to the low frequency of the descriptors “rancid aroma,” “acidic taste,” “bitter taste,” and “rancid flavor.” Additionally, anacardic acids (Section 3.1; Table 1) contribute to the characteristic cashew flavor (Oiram Filho et al., 2023).

The Cochran's *Q* test indicated that the samples differed significantly from each other in the attributes “presence of particles” and “acidic taste,” as shown by the *p* values (*p* ≤ 0.05) (Table 6). However, the McNemar test indicated no significant differences between the samples (Table 6). Therefore, the results suggest that consumers did not notice major differences in the sensory properties of the oils during storage, which is positive, as it indicates that the oil remained stable over time, even at the highest temperatures (40°C and 50°C).

**FIGURE 2:**
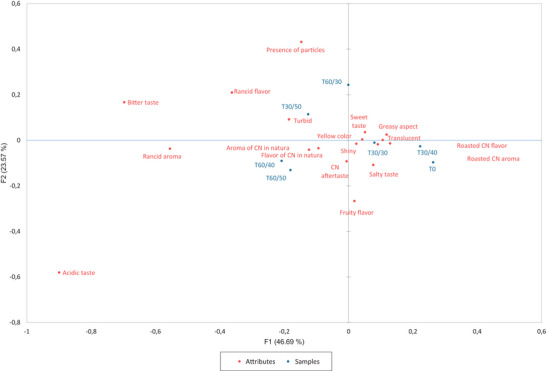
Correspondence analysis (CA) between cashew nut oil samples and Check‐All‐That‐Apply (CATA) terms.

In the CA (Figure 2), it was observed that the samples from 60 days at 40°C and 50°C (T60/40 and T60/50, respectively) were the ones most closely associated with the attributes “aroma of cashew nut *in natura*,” “flavor of cashew nut *in natura*,” “cashew nut aftertaste,” and “turbid.”

On the other hand, samples from the 0 (T0) and 30 days at 30°C and 40°C (T30/30 and T30/40) were closer to the attributes “roasted cashew nut flavor,” “roasted cashew nut aroma,” “translucent,” “greasy aspect,” “sweet taste,” “shiny,” “yellow color,” “salty taste,” and “cashew nut aftertaste.”

The attribute “rancid flavor” was more closely associated with samples stored for longer times and at higher temperatures (T30/50, T60/40, and T60/50), despite not being one of the terms most commonly used to describe these oils, which were better characterized by “sweet taste,” “yellow color,” “shiny,” and “turbid.”

**FIGURE 3:**
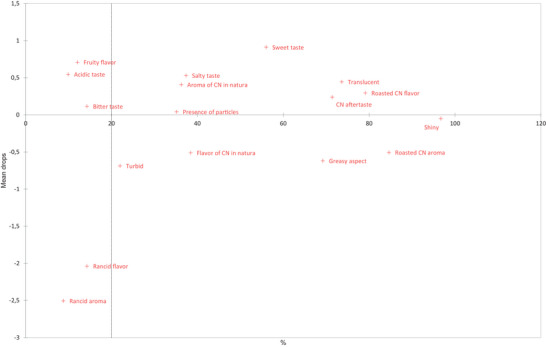
Penalty analysis (PA) presenting the impact of perceived sensory attributes on global acceptance of cashew nut oil.

Figure 3 presents the PA, which was conducted to investigate the relationship between global acceptance data and the occurrences of attributes from the CATA test, showing the drops in global acceptance averages depending on the frequency with which attributes were checked in the samples. Attributes checked with frequencies greater than 20% and influenced the global acceptance average by at least 0.60 points are considered significant. As a result, the attributes “turbid” and “greasy aspect” negatively impacted the acceptance of cashew nut oil, contributing to drops of 0.69 and 0.62 points, with frequencies of 21.98% and 69.23%, respectively. It was observed through correspondence analysis (Figure 2) that the oldest oils (T30/50, T60/40, and T60/50) became cloudier, while the freshest oils (T0, T30/30, and T30/40) exhibited a more greasy aspect.

In addition, the descriptors “flavor of cashew nut *in natura*” and “roasted cashew nut aroma” resulted in a drop of 0.51 points in both cases. The descriptors “rancid flavor” and “rancid aroma” were responsible for declines of 2.04 and 2.51 points, respectively. However, these had input frequencies lower than 20%, making them statistically insignificant (Figure 3). Still, this may have impacted the drop in global acceptance of samples stored for longer periods and at higher temperatures (T60/40, T60/50, T30/50) (Table 5; Figure 2).

The attributes “oily,” “burnt,” “bitter,” and “rancid” were negative in sesame oil (Yin et al., 2020). Similarly, the terms “buttery,” “astringent,” “pungent,” and “bitter” negatively impacted the acceptability of olive oil (Piochi et al., 2021). Therefore, descriptors referring to greasy/buttery and rancid characteristics frequently appear as undesirable in oils.

On the other hand, “sweet taste” was the descriptor that most positively influenced the general acceptance of cashew nut oil, increasing the average by 0.91 points. Other attributes that, although not statistically significant, were seen as positively influencing general acceptance included “salty taste,” “aroma of cashew nut *in natura*,” “translucent,” “roasted cashew nut flavor,” and “cashew nut aftertaste.”

Attributes such as “sweet smell,” “smooth,” “cooked sesame seed,” “nutty,” “highly intense flavor,” and “roasted” were positively correlated with sesame oil acceptability ratings (Yin et al., 2020). In olive oil, the flavors “olive,” “herbaceous,” and “sweet” were considered positive drivers of taste (Piochi et al., 2021). The “sweet” attribute seems to be a consensus as a positive characteristic in oils.

## 4 CONCLUSION

Cashew nut oil contains anacardic acids and phytosterols (mainly β‐sitosterol) and is predominantly composed of oleic acid, a monounsaturated fatty acid. During storage, subtle changes in the oil's oxidative quality were observed, particularly in samples stored at 50°C. These changes included increases in acid, peroxide, color difference values, and a reduction in the proportions of polyunsaturated fatty acids. However, the values did not exceed the limits set by the Codex Alimentarius for acid and peroxide. Sensory acceptance decreased over time, but the samples remained above the acceptance threshold even at the end of the storage period (60 days). This suggests the oil can maintain acceptable characteristics for over 60 days, even at 50°C. Additionally, the oil's sensory properties were well accepted due to its positive attributes, such as its yellow color, glossy, light appearance, sweet taste, neutral flavor, and cashew nut aroma.

Thus, the oil exhibited stable characteristics throughout storage (even at higher temperatures), demonstrating great potential for commercialization due to its favorable chemical, physical, nutritional, and sensory properties. It holds promise for direct consumption (in salads and breads) and is used as an ingredient in various culinary preparations, such as cakes, cookies, and sauces. Therefore, it is a promising product in gastronomy and nutrition.

## AUTHOR CONTRIBUTIONS

**Amanda Rodrigues Leal**: Conceptualization; investigation; data curation; writing—original draft; writing—review and editing; visualization. **Gilleno Ferreira de Oliveira**: Investigation; writing—review and editing. **Emilly Kaiane Maia da Silva**: Investigation; writing—review and editing. **Ana Jady Cavalcanti Araújo**: Investigation; writing—review and editing. **Idila Maria da Silva Araújo**: Investigation; writing—review and editing. **Hilton César Rodrigues Magalhães**: Formal analysis; investigation; writing—review and editing. **Paulo Riceli Vasconcelos Ribeiro**: Formal analysis; investigation; writing—review and editing. **Arthur Claudio Rodrigues de Souza**: Investigation; writing—review and editing. **Ana Paula Dionísio**: Conceptualization; writing—review and editing; supervision; funding acquisition; project administration. **Paulo Henrique Machado de Sousa**: Conceptualization; formal analysis; supervision; writing—review and editing.

## CONFLICT OF INTEREST STATEMENT

The authors declare no conflicts of interest.

## Supporting information



Supporting Information
