# Supplementary material for: Oxidative stability and affective/descriptive sensory properties of cashew nut (Anacardium occidentale L.) oil during accelerated storage conditions
Source: J Food Sci. 2025 Apr 9;90(4):e70176. doi: 10.1111/1750-3841.70176 (PMC11982663; doi:10.1111/1750-3841.70176)
Supplement: Supplementary file 1 — Supporting Information [file JFDS-90-0-s001.docx]

**Oxidative Stability and Affective/Descriptive Sensory Properties of Cashew Nut Oil during Accelerated Storage Conditions**

**Author(s):** Amanda Rodrigues Leal ^a,*^, Gilleno Ferreira de Oliveira^a^, Emilly Kaiane Maia da Silva^b^, Ana Jady Cavalcanti Araújo^b^, Idila Maria da Silva Araújo^b^, Hilton César Rodrigues Magalhães^b^, Paulo Riceli Vasconcelos Ribeiro^b^, Arthur Claudio Rodrigues de Souza^b^, Ana Paula Dionísio^b^, Paulo Henrique Machado de Sousa^a,c^

**Affiliation(s):**

^a^ Department of Food Engineering, Federal University of Ceara, 60356-000 Fortaleza, CE, Brazil

^b^ Embrapa Agroindústria Tropical, Dra Sara Mesquita St., 2270, 60511-110 Fortaleza, CE, Brazil

^c^ Graduate Program in Gastronomy, Culture and Art Institute, Federal University of Ceara, 60356-000 Fortaleza, CE, Brazil

*** Corresponding author:** Amanda Rodrigues Leal, Department of Food Engineering, Federal University of Ceara, Mister Hull Ave., 2977, Fortaleza, Ceara 60356-000, Brazil; E-mail address: amanda.lleal@hotmail.com.

**Table S1.** Analysis of variance of acid value, peroxide value, and color results.

| Acid value | DF | Sum of squares | Mean of squares | F | Pr > F | p-values significance codes |
| --- | --- | --- | --- | --- | --- | --- |
| Time | 1.000 | 22.014 | 22.014 | 1253.656 | **<0.0001** | *** |
| Temperature | 1.000 | 8.696 | 8.696 | 495.211 | **<0.0001** | *** |
| Time*Temperature | 1.000 | 0.263 | 0.263 | 14.989 | **0.000** | *** |
| Peroxide value | DF | Sum of squares | Mean of squares | F | **Pr > F** | p-values significance codes |
| Time | 1.000 | 180.651 | 180.651 | 1450.540 | **<0.0001** | *** |
| Temperature | 1.000 | 37.674 | 37.674 | 302.505 | **<0.0001** | *** |
| Time*Temperature | 1.000 | 8.484 | 8.484 | 68.124 | **<0.0001** | *** |
| L* | DF | Sum of squares | Mean of squares | F | **Pr > F** | p-values significance codes |
| Time | 1.000 | 246653.228 | 246653.228 | 1726.954 | **<0.0001** | *** |
| Temperature | 1.000 | 121764.676 | 121764.676 | 852.541 | **<0.0001** | *** |
| Time*Temperature | 1.000 | 11058.430 | 11058.430 | 77.426 | **<0.0001** | *** |
| a* | DF | Sum of squares | Mean of squares | F | **Pr > F** | p-values significance codes |
| Time | 1.000 | 201.535 | 201.535 | 383.731 | **<0.0001** | *** |
| Temperature | 1.000 | 168.551 | 168.551 | 320.929 | **<0.0001** | *** |
| Time*Temperature | 1.000 | 29.807 | 29.807 | 56.754 | **<0.0001** | *** |
| b* | DF | Sum of squares | Mean of squares | F | **Pr > F** | p-values significancecodes |
| Time | 1.000 | 54447.912 | 54447.912 | 1946.614 | **<0.0001** | *** |
| Temperature | 1.000 | 24695.013 | 24695.013 | 882.893 | **<0.0001** | *** |
| Time*Temperature | 1.000 | 2286.710 | 2286.710 | 81.754 | **<0.0001** | *** |

Signification codes: *** p ≤ 0,001; ** p ≤ 0.01; * p ≤ 0.05; . p ≤ 0,1; ° p ≤ 1. DF: degrees of freedom.

**Table S2.** Analysis of variance of the results from the fatty acids composition.

| Palmitic acid | DF | Sum of squares | Mean of squares | F | Pr > F | p-values significancecodes |
| --- | --- | --- | --- | --- | --- | --- |
| Time | 1.000 | 1430.168 | 1430.168 | 1212.856 | **<0.0001** | *** |
| Temperature | 1.000 | 662.091 | 662.091 | 561.487 | **<0.0001** | *** |
| Time*Temperature | 1.000 | 53.351 | 53.351 | 45.244 | **<0.0001** | *** |
| Palmitoleic acid | DF | Sum of squares | Mean of squares | F | **Pr > F** | p-values significance codes |
| Time | 1.000 | 1.470 | 1.470 | 241.600 | **<0.0001** | *** |
| Temperature | 1.000 | 0.421 | 0.421 | 69.118 | **<0.0001** | *** |
| Time*Temperature | 1.000 | 0.068 | 0.068 | 11.187 | **0.002** | ** |
| Heptadecenoic acid | DF | Sum of squares | Mean of squares | F | **Pr > F** | p-values significance codes |
| Time | 1.000 | 0.386 | 0.386 | 49.889 | **<0.0001** | *** |
| Temperature | 1.000 | 0.043 | 0.043 | 5.593 | **0.025** | * |
| Time*Temperature | 1.000 | 0.023 | 0.023 | 2.922 | **0.099** | . |
| Estearic acid | DF | Sum of squares | Mean of squares | F | **Pr > F** | p-values significance codes |
| Time | 1.000 | 1345.583 | 1345.583 | 1060.308 | **<0.0001** | *** |
| Temperature | 1.000 | 660.293 | 660.293 | 520.305 | **<0.0001** | *** |
| Time*Temperature | 1.000 | 54.579 | 54.579 | 43.008 | **<0.0001** | *** |
| Oleic acid | DF | Sum of squares | Mean of squares | F | **Pr > F** | p-values significance codes |
| Time | 1.000 | 86905.316 | 86905.316 | 1388.860 | **<0.0001** | *** |
| Temperature | 1.000 | 38047.338 | 38047.338 | 608.046 | **<0.0001** | *** |
| Time*Temperature | 1.000 | 2989.825 | 2989.825 | 47.781 | **<0.0001** | *** |
| Linoleic acid | DF | Sum of squares | Mean of squares | F | **Pr > F** | p-values significance codes |
| Time | 1.000 | 5235.251 | 5235.251 | 1085.350 | **<0.0001** | *** |
| Temperature | 1.000 | 2455.003 | 2455.003 | 508.961 | **<0.0001** | *** |
| Time*Temperature | 1.000 | 197.991 | 197.991 | 41.047 | **<0.0001** | *** |
| Arachidonic acid | DF | Sum of squares | Mean of squares | F | **Pr > F** | p-values significance codes |
| Time | 1.000 | 6.776 | 6.776 | 77.402 | **<0.0001** | *** |
| Temperature | 1.000 | 0.501 | 0.501 | 5.722 | **0.024** | * |
| Time*Temperature | 1.000 | 0.043 | 0.043 | 0.491 | **0.489** | ° |
| Unidentified | DF | Sum of squares | Mean of squares | F | **Pr > F** | p-values significance codes |
| Time | 1.000 | 1.278 | 1.278 | 43.223 | **<0.0001** | *** |
| Temperature | 1.000 | 0.308 | 0.308 | 10.418 | **0.003** | ** |
| Time*Temperature | 1.000 | 0.076 | 0.076 | 2.584 | **0.120** | ° |
| Saturated fatty acids | DF | Sum of squares | Mean of squares | F | **Pr > F** | p-values significance codes |
| Time | 1.000 | 5550.214 | 5550.214 | 1159.874 | **<0.0001** | *** |
| Temperature | 1.000 | 2644.765 | 2644.765 | 552.698 | **<0.0001** | *** |
| Time*Temperature | 1.000 | 215.852 | 215.852 | 45.108 | **<0.0001** | *** |
| Monounsaturated fatty acids | DF | Sum of squares | Mean of squares | F | **Pr > F** | p-values significance codes |
| Time | 1.000 | 87989.900 | 87989.900 | 1391.613 | **<0.0001** | *** |
| Temperature | 1.000 | 38382.233 | 38382.233 | 607.038 | **<0.0001** | *** |
| Time*Temperature | 1.000 | 3034.973 | 3034.973 | 48.000 | **<0.0001** | *** |
| Polyunsaturated fatty acids | DF | Sum of squares | Mean of squares | F | **Pr > F** | p-values significance codes |
| Time | 1.000 | 5618.713 | 5618.713 | 1256.222 | **<0.0001** | *** |
| Temperature | 1.000 | 2525.640 | 2525.640 | 564.678 | **<0.0001** | *** |
| Time*Temperature | 1.000 | 203.870 | 203.870 | 45.581 | **<0.0001** | *** |

Signification codes: *** p ≤ 0,001; ** p ≤ 0.01; * p ≤ 0.05; . p ≤ 0,1; ° p ≤ 1. DF: degrees of freedom.

**Table S3.** Analysis of variance of sensory acceptance results.

| Appearance | DF | Sum of squares | Mean of squares | F | Pr > F | p-values significancecodes |
| --- | --- | --- | --- | --- | --- | --- |
| Time | 1.000 | 3618.462 | 3618.462 | 1619.530 | **<0.0001** | *** |
| Temperature | 1.000 | 2674.370 | 2674.370 | 1196.979 | **<0.0001** | *** |
| Time*Temperature | 1.000 | 160.462 | 160.462 | 71.819 | **<0.0001** | *** |
| Aroma | DF | Sum of squares | Mean of squares | F | **Pr > F** | p-values significance codes |
| Time | 1.000 | 3635.713 | 3635.713 | 1532.488 | **<0.0001** | *** |
| Temperature | 1.000 | 2554.173 | 2554.173 | 1076.609 | **<0.0001** | *** |
| Time*Temperature | 1.000 | 171.658 | 171.658 | 72.355 | **<0.0001** | *** |
| Flavor | DF | Sum of squares | Mean of squares | F | **Pr > F** | p-values significance codes |
| Time | 1.000 | 3627.082 | 3627.082 | 1507.264 | **<0.0001** | *** |
| Temperature | 1.000 | 2531.025 | 2531.025 | 1051.788 | **<0.0001** | *** |
| Time*Temperature | 1.000 | 194.563 | 194.563 | 80.852 | **<0.0001** | *** |
| Global acceptance | DF | Sum of squares | Mean of squares | F | **Pr > F** | p-values significancecodes |
| Time | 1.000 | 3609.851 | 3609.851 | 1552.051 | **<0.0001** | *** |
| Temperature | 1.000 | 2467.739 | 2467.739 | 1061.001 | **<0.0001** | *** |
| Time*Temperature | 1.000 | 190.262 | 190.262 | 81.803 | **<0.0001** | *** |

Signification codes: *** p ≤ 0,001; ** p ≤ 0.01; * p ≤ 0.05; . p ≤ 0,1; ° p ≤ 1. DF: degrees of freedom.
